# Pulmonary Sclerosing Pneumocytoma: An Essential Differential Diagnosis for a Lung Nodule

**DOI:** 10.7759/cureus.21081

**Published:** 2022-01-10

**Authors:** Rajapriya Manickam, Ashesha Mechineni

**Affiliations:** 1 Pulmonary/Critical Care Medicine, St. Joseph's University Medical Center, Paterson, USA; 2 Internal Medicine, St. Joseph's University Medical Center, Chicago, USA

**Keywords:** pulmonary lobectomy, lung tumor, lung nodule, hemangioma, pneumocytoma

## Abstract

Pulmonary sclerosing pneumocytoma, previously known as pulmonary sclerosing hemangioma, is a rare benign lung tumor with a low prevalence. We present this condition in a 26-year-old, young, non-smoking female with a slow-growing pulmonary nodule incidentally noted on imaging. Serial computed tomography(CT) scans revealed slow growth, and invasive testing was recommended. The patient underwent a left lateral thoracotomy and based on frozen section findings. A left lower lobectomy was performed. The final pathological diagnosis revealed sclerosing pneumocytoma. This is an atypical patient demographic considering the propensity of the disease for middle-aged Asian women. The case presentation and work-up highlight this critical differential diagnosis for incidental pulmonary nodules increasingly being noted due to widespread use of imaging for screening and routine care in the current medical climate. There are no specific imaging criteria to diagnose this condition. The final diagnosis is made only after surgical biopsy and histopathology. No additional treatment is needed following the diagnosis.

## Introduction

Pulmonary Sclerosing Pneumocytoma (PSP) was first described by Liebow and Hubbell in 1956 [1]. Though there were many controversies regarding the origin of the tumor, recent immunohistochemical studies strongly suggest an origin from respiratory epithelium, particularly type II alveolar pneumocytes. In the most recent update, World Health Organization (WHO) reclassified this lung tumor from miscellaneous group to adenomas/epithelial tumors [2]. Publication of Fleishner Society guidelines heralded the increased diagnosis and evaluation of incidental pulmonary nodules. Reports indicate increased imaging has led to a higher prevalence of detected benign pulmonary nodules [3]. High-value care dictates we evaluate incidental pulmonary nodules in risk factors and patient demographic information. This case report presents an incidental pulmonary nodule finding in a low-risk individual patient.

## Case presentation

**Figure 1:**
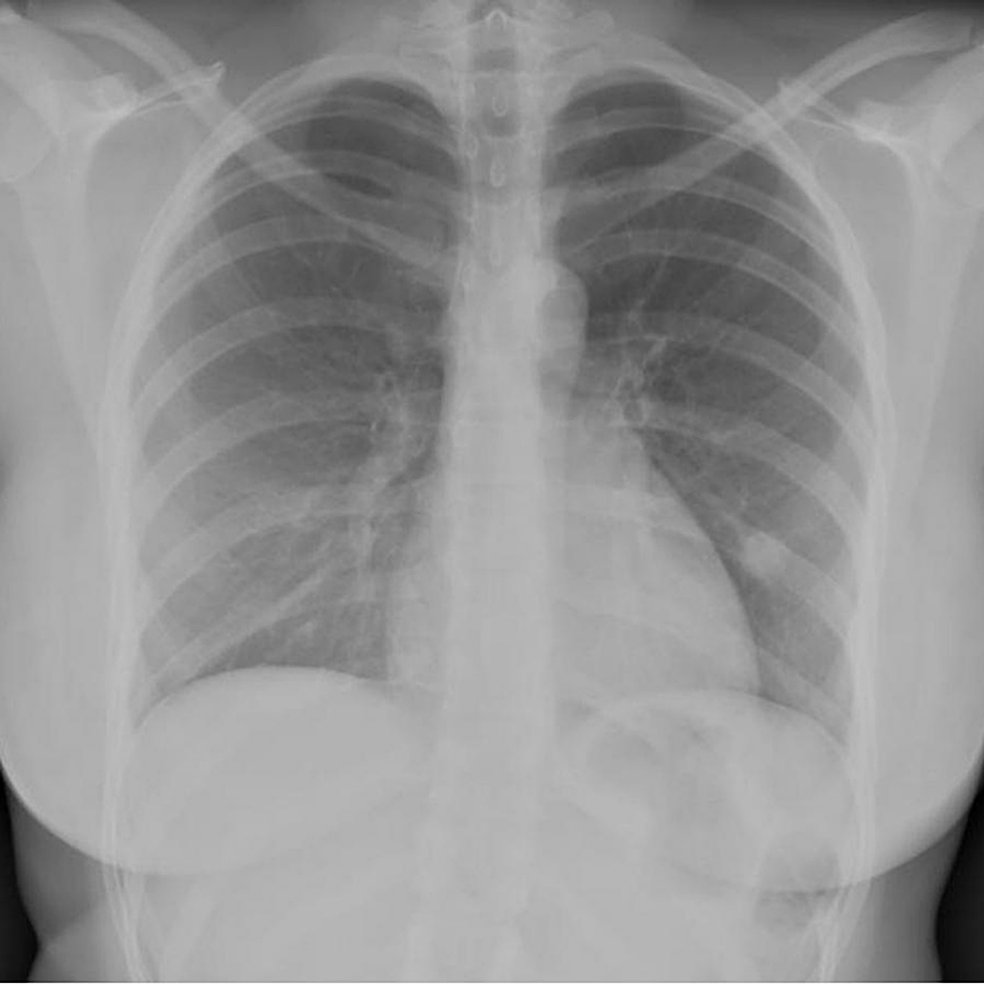
Chest radiograph of the patient showing a pulmonary nodule. The smooth, non-cavitating, non-calcified features of the nodule can be appreciated.

A 26 years old Hispanic female with a medical history of bronchial asthma was referred to the pulmonary clinic to evaluate a 1 cm solitary pulmonary nodule noted on a routine chest radiograph (figure 1).

She is a non-smoker with no history of recent travel. The patient reported only occasional productive cough; denied any history of dyspnea, chest pain, hemoptysis, fever, chills, night sweats, or weight loss. She denied any sick contacts or pets or a history of malignancy in her family. The nodule was initially reported on a Chest X-ray about one year before referral. Still, the patient did not follow up with a Computed tomography (CT) of the thorax as advised. A subsequent CT scan of the chest confirmed a 1 cm soft tissue non-calcified, a non-cavitating nodule in the basilar segment of the left lower lobe. There were no other abnormal findings.

**Figure 2:**
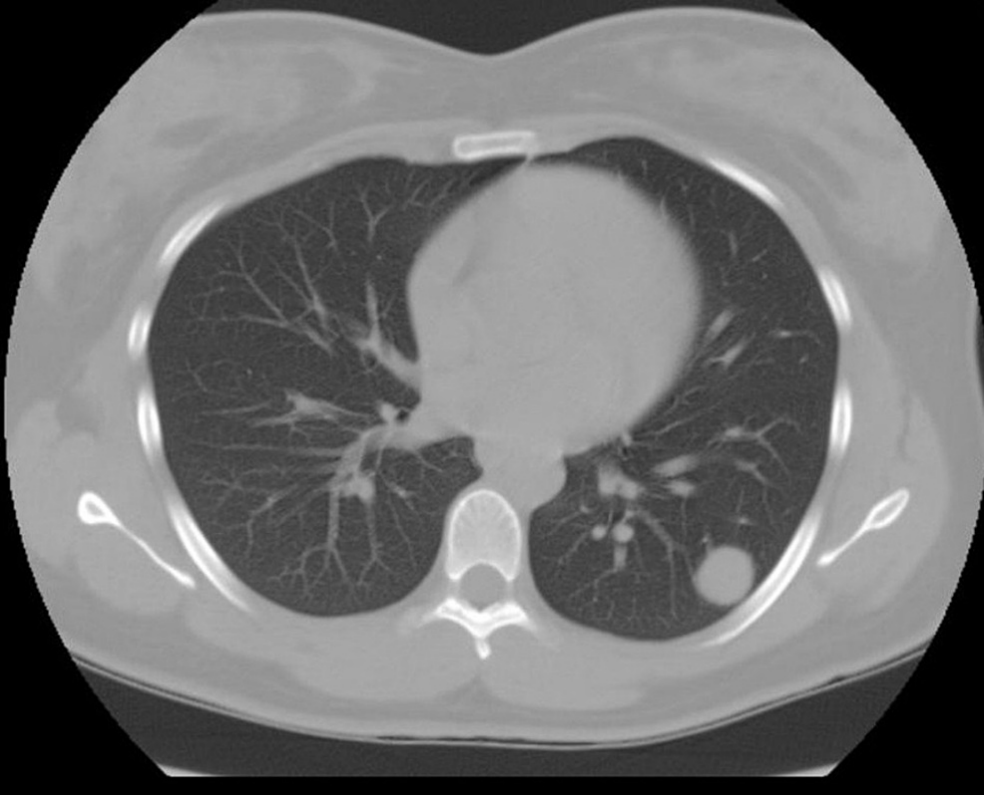
Computed tomography of chest showing pulmonary nodule. Even with the growth of nodule from its first diagnosis, it retains the non-cavitary and non-calcified features. The peripheral location of the nodule in the basilar segment of the left lower pulmonary lobe is appreciated.

Given the benign-looking features of the nodule, stability in size, and low-risk profile of the patient, she was advised expectant follow-up with further guideline-recommended imaging. She had serial CT scans for six years which showed a gradual increase in size, and the nodule was 2cm in diameter on the last CT scan (figure 2). 

**Figure 3:**
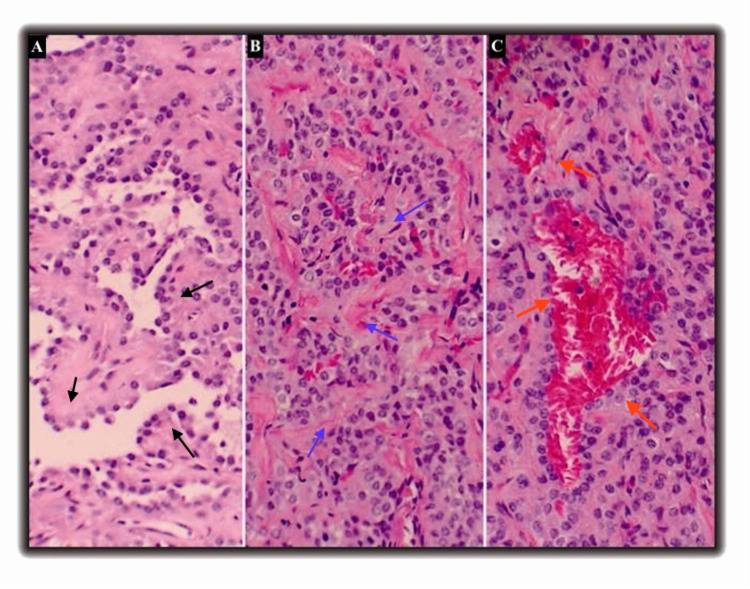
Patient biopsy sample revealing papillary (black arrows), solid(blue arrows), and hemorrhagic (red arrows) morphologies associated with PSP. Light microscopy images show papillary (panel A), solid (panel B), and hemorrhagic patterns (panel C) identified in the three different parts of the image. PSP: Pulmonary Sclerosing Pneumocytoma

Her physical examination was unremarkable. Laboratory findings including white cell count, hemoglobin, renal function panel, hepatic function panel, coagulation parameters, erythrocyte sedimentation rate (ESR), rheumatoid factor were normal. Pulmonary function test revealed no obstructions but moderate restriction with normal diffusing capacity for carbon monoxide (DLCO). Further invasive testing included a CT-guided trans-thoracic biopsy, which was non-diagnostic, and the patient was referred to a surgical wedge biopsy. She underwent a left lateral thoracotomy, and the nodule was resected. Frozen section examination suggested adenocarcinoma and hence left lower lobectomy was performed. No lymph nodes were found during exploration. The subsequent pathological evaluation showed sclerotic papillary, solid, and hemorrhagic growth patterns with dilated blood-filled spaces suggesting PSP diagnosis (figure 3).

Epithelial membrane antigen (EMA) and antibodies to thyroid transcription factor-1 (TTF-1) were positive, leading to a diagnosis of Pulmonary Sclerosing Pneumocytoma. The patient remains stable at six months and one-year follow-up and has no further clinical complications or recurrence.

## Discussion

Though reported in younger age groups like our patient, PSP typically presents in the fifth decade, with high female preponderance (female: male ratio of 5:1) [4] and more common in Asia. Most patients are asymptomatic (50-70%), with tumors incidentally detected on chest imaging. Symptomatic patients were commonly present with hemoptysis, cough, or chest pain [5]. 

On the chest radiograph, PSP is seen as a solitary, peripheral, well-defined, homogenous nodule. CT chest is seen as round or oval juxta pleural nodule or mass, mostly 2-3 cm in diameter with smooth margins, with occasional peripheral calcification (13%). Few multiple or bilateral lesions are also reported [6]. The tumor shows marked enhancement with contrast (mean 79HU after contrast) [7], usually homogenous, but large tumors can have heterogeneous enhancement. A rare but interesting finding on the CT scan is the “air meniscus sign,” which is described as a crescentic radiolucency around lung nodule postulated to be secondary to air trapping or peritumoral hemorrhage. Air crescent signs can be seen with aspergilloma, carcinoids, hamartoma, hemangioma, malignant teratoma, arterio-venous malformations, and inflammatory lesions. The tumor rarely cavitates, but a single report of PSP mimicking hydatid cyst [8]. There is no one specific or diagnostic radiologic characteristic for PSP. The majority of the PSP show increased 18F-fluorodeoxyglucose (FDG) uptake on positron emission tomography imaging [9]. Large tumors (>2cm) have high uptake and lead to misdiagnosis of malignancy.

On gross examination, PSP lesions are well-circumscribed, firm tan mass usually of 0.3-7cm size, with focal areas of hemorrhage, usually located close to the pleural surface or fissure and occasionally present as endobronchial or polypoid mass [10]. PSP on microscopic examination is characterized by two types of epithelial cells - cuboidal surface cells and round stromal cells in four distinct architectural patterns - papillary, sclerotic, solid, and hemorrhagic. The cuboidal cells line the surface of the tumor, have abundant lamellar bodies, similar to type II pneumocytes, and are called lining cells. The uniform, oval, or polygonal-shaped cells with clear cytoplasm and fine chromatin distributed in the interstitium are called round cells. Both cells rarely show cytologic atypia. PSP’s are usually composed of 2-3 architectural patterns though one pattern predominates. Other common findings are chronic inflammation, xanthomatous histiocytes, cystic spaces, cholesterol clefts, etc., which in combination gives histological heterogeneity. On immunohistochemical studies, both cells express thyroid transcription factor 1(TTF-1) and epithelial membrane antigen (EMA). The surface cells also exhibit Pan cytokeratin antibodies (AE1/AE3) and surfactant proteins A and B, indicating that they are more differentiated and originate from type II pneumocytes. In contrast, the round cells lack these proteins and express cytokeratin 7 and CAM 5.2, consistent with a primitive respiratory epithelial origin.

The peripheral location of the tumor favors CT-guided biopsy than bronchoscopy-guided biopsy. There are only a few reports of cytological findings, and the epithelial cells by themselves are difficult to differentiate from the adenocarcinoma in situ (Bronchoalveolar carcinoma). Presence of dual cell population, clusters, and papillae of nondescript, bland-looking mononuclear cells, hyalinized stromal tissue fragments, epithelial cells with scattered pleomorphism and intranuclear inclusions, foamy hemosiderin-laden macrophages, blood lakes separated by septae of tumor cells, absence of significant cytological atypia and mitotic figures can give a clue to the diagnosis. Fine needle aspiration is usually non-diagnostic; additional cell block and immunohistochemical evaluation are needed for diagnosis. Wedge resection is curative and diagnostic for PSP. Identification of 3-4 architectural patterns on the frozen section is diagnostic but challenging due to the frozen section artifact, and the reported rate of misdiagnosis is about 25% [11]. Patients end up getting unnecessary extensive surgery, which happened with our patients. When a papillary pattern predominates, samples of the tumor obtained by needle biopsy or frozen section can be confused with an epithelioid hemangioendothelioma, carcinoid tumor, or well-differentiated papillary adenocarcinoma [12].

It is essential to differentiate PSP from malignant and metastatic tumors as limited resection is curative for PSP instead of aggressive therapy for malignant lesions. Common differential malignant tumors are bronchoalveolar carcinoma, metastatic papillary thyroid carcinoma, carcinoid, and metastatic renal cell carcinoma [5,13]. Other benign tumors that should be considered in the differential diagnosis are clear cell tumors, hemangioma, and hamartomas [14,15]. The typical nuclear features and immunohistochemistry for thyroglobulin differentiate papillary thyroid carcinoma from PSP, nuclear atypia, striking vascularity sets apart metastatic renal cell carcinoma, organized ribbon-like growth patterns, and immunohistochemistry for neuroendocrine markers distinguish carcinoid from PSH. Clear cell tumors have scant stroma and have an affinity for HMB-45 expression. Hamartomas have cartilage, myxoid stroma adipose tissue in various combinations, and true hemangiomas lack epithelial cells and are hence differentiated from PSP.

PSP is benign, and surgical excision is curative without adjuvant therapy. PSP is a benign tumor, but cases of lymph nodal metastasis, especially to mediastinal and hilar nodes, have been reported. However, no hematogenous spread has been reported so far [16]. From the reported cases, the nodal metastasis or presence of multiple or bilateral lesions does not affect the long-term prognosis. Literature suggests a negligible recurrence rate, and no follow-up imaging is suggested [5,13-15].

## Conclusions

PSP is a rare benign neoplasm of adult females, which rarely metastasis to lymph nodes. Patients are usually asymptomatic, presenting with a solitary, smooth, juxta pleural pulmonary nodule found incidentally. There can be occasional calcification, with marked homogenous contrast enhancement on a CT scan and high 18 fluorodeoxyglucose uptake on a PET scan. Though the benign nature of a tumor can be ascertained by the radiological picture and slow growth rate, histopathology confirms the diagnosis. CT-guided fine-needle aspiration is the commonly opted diagnostic approach due to the peripheral location of the nodule. However, a cytological study can easily mislead well-differentiated adenocarcinoma; hence, cell blocks with immunohistochemical stains are needed for diagnosis. Histopathological examination is characterized by typical cuboidal surface cells and underlying round stromal cells with at least 2/4 architectural pattern, and immunohistochemical studies show both surface and stromal cells positive for epithelial markers, EMA, and TTF-1 while only surface cells positive for pan-cytokeratin antibodies and surfactant proteins. Limited surgical excision is curative. No additional treatment is required, even in those with multiple bilateral lesions or lymph node metastasis.
